# Increased serum procalcitonin levels in pregnant patients with asymptomatic bacteriuria

**DOI:** 10.1186/1476-0711-12-25

**Published:** 2013-09-05

**Authors:** Filiz Bilir, Nermin Akdemir, Selcuk Ozden, A Serhan Cevrioglu, Cemil Bilir

**Affiliations:** 1 Gynecology and Obstetrics, School of Medicine, Sakarya University, Sakarya 54055, Turkey; 2 Internal Medicine, School of Medicine, Bülent Ecevit University, Zonguldak 67100, Turkey

**Keywords:** Asymptomatic bacteriuria, Procalcitonin, Pregnant women, Inflammatory markers

## Abstract

**Background:**

Among the pregnancy urinary tract infections, asymptomatic bacteriuria (ASB) is the most common one. Untreated ASB can progress to pyelonephritis in 30-50% of the patients and can also result in prematurity in 27% of the pregnancy so it needs immediate diagnosis and treatment. In this study, we wanted to evaluate procalcitonin levels, compared to other inflammatory in pregnant women with ASB.

**Methods:**

The study was designed between the period of January 2012 and February 2013 at Sakarya University School of Medicine, Department of Gynecology and Obstetrics. The study population included 30 pregnant patients with asymptomatic bacteriuria and 39 healthy pregnant controls.

**Results:**

Mean age was 28 (SD, 5.5) of the study population; mean maternal weight was 70 (SD, 8) kilogram. There were no statically significant differences between the groups according to the routine biochemical parameters, but gestational age was significantly lower in the ASB group compared to the controls (20.4 vs 28.6, respectively; p < 0.001). Serum procalcitonin levels were negative in all of the controls. In ASB group, 9 (30%) patients had procalcitonin levels greater than >0.05 ng/ml and 21(70%) patients had negative procalcitonin levels (Chi-squrae, p < 0.001). The sensitivity and specificity of procalcitonin assay for ASB was calculated as 30% and 100%, respectively. The positive predictive value was 100% and the negative predictive value was 65%. The most frequent microorganisms in the urine culture were Escherichia coli (26 patients, 87%), Proteus mirabilis (3 patients, 10%) and Klebsiella (1 patient, 3%) in the ASB group. We experienced four (44%) recurrences among nine positive procalcitonin in ASB patients after completion of treatment of the first ASB diagnosis.

**Discussion:**

Procalcitonin levels were significantly higher in ASB group than the control group and serum procalcitonin levels were higher in pregnant women with recurrent ASB. This finding is an important result revealed that high procalcitonin level can predict the further urinary tract infection risk. Finally, serum procalcitonin levels were normal in healthy pregnant women while other inflammatory markers such as WBC, ESR and CRP levels were higher.

## Background

Urinary tract infections (UTI) are the most common infections during pregnancy and can cause serious maternal and fetal complications if led untreated [1]. Among the pregnancy UTIs, asymptomatic bacteriuria (ASB) is the most common one. Changing with region and race, the prevalence of ASB in pregnancy is 2,5-15%. Untreated ASB can progress to pyelonephritis in 30-50% of the patients and can also result in prematurity in 27% of the pregnancy. As the treatment of ASB reduces both the risk acute pyelonephritis and low birth weight, early diagnosis is of paramount importance [2].

Procalcitonin is a precursor of calcitonin which is known as calcium-regulating hormone and also a marker of inflammatory infections, it releases from organs other than the thyroid [3]. Procalcitonin increases especially in bacterial infections and rises faster than the commonly used inflammatory markers such as erythrocyte sedimentation rate (ESR), C reactive protein (CRP) and decreases more quickly following alleviation of the infection; Thus, it is easy and applicable in follow up the severity of infections [4].

In this study, we wanted to evaluate procalcitonin levels, compared to other inflammatory markers at standard screening visit for ASB and whether there was a difference between pregnant with and without ASB. We also analyzed the predictive and prognostic value of procalcitonin in ASB.

## Materials and methods

The study was designed between the period of January 2012 and February 2013 at Sakarya University School of Medicine, Department of Gynecology and Obstetrics and the study was approved by the local Ethical Committee for Scientific Studies at Sakarya University, Sakarya, Turkey. The study population included 30 pregnant patients with asymptomatic bacteriuria and 39 healthy pregnant controls. ASB is defined as two consecutive voided urine specimens with isolation of the same bacterial strain in quantitative counts of ≥10^5^ cfu/mL without any symptoms such as dysuria, incontinance, urgency, colic pain and fever. All women were between the ages of 18-45 and pregnancies were greater than 11 weeks of gestation. The exclusion criteria were: active sign of infection such as fever, cough, sputum, dysuria, diarrhea, etc., history of hospitalization during last month, operation or trauma history, antibiotic usage during pregnancy, any chronic illness history such as diabetes mellitus, hypertension, coronary heart disease, autoimmune disorder, pre-eclampsia or eclampsia.

Urine culture, serum CRP levels, complete blood count (CBC) and differential, ESR, serum procalcitonin and serum biochemistry were analyzed in all patients.

### Urine culture

All patients gave a morning mid-stream urine sample following the cleaning of the periurethral region. Patients with positive urine cultures gave a second a urine sample for culture in the next 48 hours. If the second result was also positive the patient was included in the ASB group of study. During the following period we order a urine microscopy and culture for each month if a patient treated for ASB at the beginning. All analyses were done in the microbiology laboratory.

### Measurement of serum procalcitonin

All serum samples were analyzed with mini-VIDAS NO: 99,737 device (bioMérieux, Inc. NC 27,712, U.S.A.) at the microbiology clinic. Serum procalcitonin levels greater than 0.05 ng/ml were quantified; levels <0.05 ng/ml were interpreted as negative.

### Statistical analysis

Continuous variables were tested by Kolmogorov-Smirnov for normality test. Normally distributed continuous variables were analyzed with two-sample t-test and abnormally distributed variables were analyzed with Mann–Whitney U test. Categorical variables were analyzed by chi-square test. All P-values less than 0.05 were considered statistically significant. Calculations were performed with statistical software (SPSS 18.0 statistical software program SPSS, Chicago, IL).

## Results

Mean age was 28 (SD, 5.5) of the study population; mean maternal weight was 70 (SD, 8) kilogram. There were no statically significant differences between the groups according to the routine biochemical parameters, but gestational age was significantly lower in the ASB group compared to the controls (20.4 vs 28.6, respectively; p < 0.001); Also, parity and gravity were significantly higher in the ASB group compared to the controls (p = 0.002 and 0.017, respectively). In terms of inflammatory markers, ESR and CRP levels were similar (Table 1 and 2).

**Table 1:** The general characteristics of asymptomatic bacteriuria and control groups

	**Control (n = 39)**	**Asymptomatic bacteriuria group (n = 30)**	**P**
**Age**	29,36 ± 5,91	26,97 ± 4,68	0.073
**Weight**	70 [65–76]	68 [66,5-72]	0.799
**Gestational age**	28,64 ± 8,74	20,4 ± 5,98	0.0001
**CRP**	4,98 [3–8,35]	7,79 [4,56-8,9]	0.066
**WBC**	9682,05 ± 2468,31	8454,33 ± 2716,41	0.054
**Hb, gr/dl**	10,8 ± 1.65	11,9 ± 2.12	0,95
**AST, U/L**	25 ± 4.23	20 ± 6.25	0.7
**ALT,U/L**	21 ± 3.22	19 ± 4.55	0.5
**Cre, mg/dl**	0.7 ± 0.15	0.8 ± 0.19	0.8
**Sedim 1**	16 [11–23]	22 [9–23]	0.780
**Sedim 2**	27 [20–39]	35 [15–37]	0.908

CRP: C reactive protein, WBC: white blood cell, Hb:Hemoglobulin, AST: Aspartate transaminase, ALT: Alanin transaminase, Cre: Creatinine.

P values were <0.05 accepted as statically significant.

**Table 2:** Pregnancy outcomes of the study population

	**Control (n = 39)**	**Asymptomatic bacteriuria group (n = 30)**	**Pearson chi-square**	**P value (Chi-Sq)**	**P value(Yates value)**
**Gravida**	2 [1–3]	2 [2–4]	16.7	0.004	0.07
**Parity**	1 [0–1]	1 [1–3]	11.7	0.008	0.028
**Abortus**	0 [0–1]	0 [0–1]	4.1	0.12	0.36
**Live birth**	1 [0–1]	1 [1–2]	8.7	0.033	0.11

P values of pregnancy outcomes were analyzed by Chi-Square and Yates correction.

P values were <0.05 accepted as statically significant.

**Figure 1:**
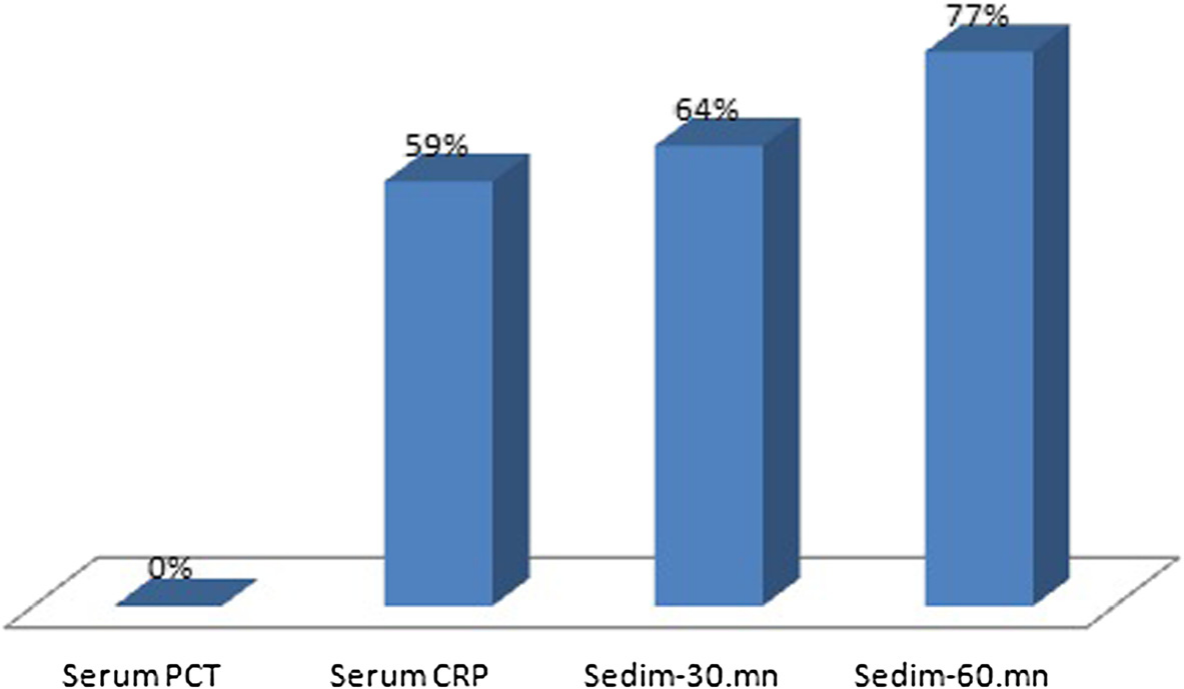
Serum Procalcitonin, CRP, WBC and sedimentation levels of healthy control group.

Urine analyses were similar between the groups but leukocyte count and bacteriuria in the microscopic analyses was significantly higher in the ASB group compared to the controls (p < 0.001 and 0.0015, respectively). Serum procalcitonin levels were negative in all of the controls. Serum prevalence of CRP, WBC and ESR elevations in healthy control group were illustrated in the Figure 1.

In ASB group, 9 (30%) patients had procalcitonin levels greater than >0.05 ng/ml and 21(70%) patients had negative procalcitonin levels (Chi-squrae, p < 0.001) (Table 3).

**Table 3:** Serum Procalcitonin levels in ASB and control groups

	**Control (n = 39)**	**ASB group (n = 30)**	**P**
**Procalcitonin (+)**	0	9 (30.0)	0.0009
**Procalcitonin (−)**	39	21	

OR = 2.857 (%95 CI: 2.024-4.032). P values were <0.05 accepted as statically significant.

The sensitivity and specificity of procalcitonin assay for ASB was calculated as 30% and 100%, respectively. The positive predictive value was 100% and the negative predictive value was 65%.

The most frequent microorganisms in the urine culture were Escherichia coli (26 patients, 87%), Proteus mirabilis (3 patients, 10%) and Klebsiella (1 patient, 3%) in the ASB group. We experienced four (44%) recurrences among nine positive procalcitonin in ASB patients after completion of treatment of the first ASB diagnosis. In all recurrences urine culture were positive for Escherichia coli as the initial ASB.

## Discussions

We found three important results in this study which investigated serum procalcitonin levels in pregnant women with ASB. The first one: Serum procalcitonin levels are significantly higher in pregnant women with ASB than the control group. The second one is: Recurrent ASB and urinary tract infection risk are higher in pregnant women with positive procalcitonin levels at the time of first diagnosis of ASB. This result revealed that high procalcitonin level might be a risk factor for subsequent complications in pregnant women with ASB and the third result of the study is procalcitonin level was normal despite the higher levels of WBC, ESR and CRP levels in the control group. These results supported that serum procalcitonin is a reliable marker for either diagnoses or exclusion of urinary tract infection in pregnant women.

ASB is the most common type of urinary tract infection in pregnancy and found in 4-7% of pregnancies [5]. Pregnancy may cause anatomical, physiological and hormonal changes in women and these changes can increase the frequency of ASB [6]. Presence of ASB in a pregnant woman represents a significant health risk. The most important risk is development of acute and chronic pyelonephritis in pregnant women with ASB. It is known that pyelonephritis can develop in 15-50% of pregnant women if ASB is not treated [7]. Many complications can be prevented with early treatment of ASB during pregnancy. In this respect, studies for early diagnosis and treatment of ASB during pregnancy are very important.

It is accepted that, routine screening of pregnant women for ASB, should be done in the first visit and at regular intervals during pregnancy for early diagnosis and treatment but some authors suggest the screening of ASB in regions with high prevalance [8,9]. Our region is not a high prevalence area for ASB so most of patients were in the second trimester in our study. Urine culture is still the screening method for ASB. However it takes time, a second sample for culture is necessary for definitive diagnosis and it does not predict recurrences.

Serum procalcitonin is a new marker in pregnant women population. There was no significant difference of serum procalcitonin levels between pregnant women and healthy controls [8,10,11]. Procalcitonin levels do not change with pregnancy-related conditions (type of birth, time of birth, type of anesthesia and the stress of childbirth), but are affected by premature rupture of membranes and infectious conditions, such as group B streptococcal colonization [12]. Montagnana et al. showed that serum procalcitonin was associated with preeclampsia [13]. Following this study, Can et al. found that there was a significant correlation between severe preeclampsia and procalcitonin levels [5]. Another study published recently showed that serum procalcitonin, CRP and D-dimer was significantly associated with preeclampsia [14].

To our knowledge, this is the first study in the literature investigating serum procalcitonin levels in pregnant women with ASB; thus, normal limit of serum procalcitonin levels in pregnant women with ASB is not established yet. Our study showed that 30% of the patient with ASB had high procalcitonin levels and all patients in the control group had negative procalcitonin assays. We calculated the sensitivity and specificity of procalcitonin assay for ASB as 30% and 100%, respectively furthermore, positive predictive value was 100% and the negative predictive value was 65%. Therefore we suggest that procalcitonin may be used in conjunction with urine culture in ASB diagnosis and follow up.

Serum procalcitonin level has still been studied in urinary tract infections and a significant correlation between the grade 3 vesico-urethral reflux and procalcitonin was shown in the children. In the same study, serum procalcitonin levels which were higher than 0, 5 ng/ml were associated with an increased risk of recurrent urinary tract infection [15]. Consistent with this study, we found significant recurrence rate in ASB patients with elevated procaltionin levels. In our study 44% of ASB patients had recurrence following the 2 months of the treatment of ASB and all of them had elevated procalcitonin levels. In another study among 1-13 months old babies, serum procalcitonin levels predicted acute pyelonephritis complicated with renal scar with 83% sensitivity and 94% specificity [16]. In our study, none of the women experienced systemic infection or sepsis and we don’t have fetal outcome data.

Other studies were performed investigating the predictive value of serum procalcitonin levels in urinary tract infections of children, but there was limited number of studies about pregnant women especially with urinary tract infections.

In our study, positive of serum assay was detected in 30% of pregnant women with ASB, and 4 of these 9 patients (44%) developed recurrent urinary tract infections after two months of treatment. This result supported that positive serum procalcitonin assay could be a predictive marker of recurrent urinary tract infections in the ASB population.

For the other parameters for infections: White blood cell count is usually normal in pregnancy, but the number of neutrophils can increase. There might be a significant leucocytosis increased up to 25.000 / mm3 during labor and puerperium. Also serum alkaline phosphatase can increase and may associate with inflammation [17]. In our study, leucocytosis was not seen in pregnant women as in controls. ESR starts to increase after 10 th weeks of gestation. The increase is moderate and it returns to normal level at 1 month after labor [18]. CRP levels were shown to be significantly higher in healthy pregnant women during the first 4 weeks of gestation [19]. In our study, CRP and ESR levels were above normal, but there was no significantly difference between the control and ASB group. But none of the healthy pregnant women had elevated procalcitonin levels and there was significant elevation of procalcitonin in the ASB group. This result showed that serum procalcitonin assay might be more reliable inflammatory marker than ESR, WBC and CRP in pregnant women with ASB.

## Conclusions

In conclusion, this is the first study that investigated the serum procalcitonin levels in pregnant women with ASB. Procalcitonin levels were significantly higher in ASB group than the control group. Also serum procalcitonin levels were higher in pregnant women with recurrent ASB. This finding is an important result revealed that high procalcitonin level can predict the further urinary tract infection risk. Finally, serum procalcitonin levels were normal in healthy pregnant women while other inflammatory markers such as WBC, ESR and CRP levels were higher. Finally we can suggest that serum procalcitonin assay may be a reliable marker for diagnosis and exclusion of urinary tract infections in pregnant women.

## Competing interests

The authors declare that they have no competing interests.

## Authors’ contributions

FB: designed the study and did the acquisition of the data. NA: drafting the manuscript. SO and ASC: revised the article and given final approval. CB: analysis and interpretation of data. All authors read and approved the final manuscript.

## References

[B1] Mittal P Wing DA Urinary tract infections in pregnancy Clin Perinatol 2005 32 749 7641 10.1016/j.clp.2005.05.006 16085031

[B2] Millar LK Cox SM Urinary tract infections complicating pregnancy Infect Dis Clin North Am 1997 11 13 26 10.1016/S0891-5520(05)70339-1 9067782

[B3] Harbarth S Holeckova K Froidevaux C Pittet D Ricou B Grau GE Vadas L Pugin J Geneva sepsis network: diagnostic value of procalcitonin, interleukin-6, and interleukin-8 in critically ill patients admitted with suspected sepsis Am J Respir Crit Care Med 2001 164 396 402 10.1164/ajrccm.164.3.2009052 11500339

[B4] Schneider HG Lam QT Procalcitonin for the clinical laboratory: a review Pathology 2007 39 383 390 10.1080/00313020701444564 17676478

[B5] Can M Sancar E Harma M Guven B Mungan G Acikgoz S Inflammatory markers in preeclamptic patients Clin Chem Lab Med 2011 49 1469 1472 21913791 10.1515/CCLM.2011.232

[B6] Arıgüloğlu EA Ayanoğlu A Numanoğlu C Altuncu N Ceylan Y Gebelikte asemptomatik bakteriüri sıklığı Perinatoloji dergisi 1994 2 231 234

[B7] Faro S Fenner DE Urinary tract infections Clin Obstet Gynecol 1998 41 744 754 10.1097/00003081-199809000-00030 9742370

[B8] Assicot M Gendrel D Carsin H Raymond J Guilbaud J Bohuon C High serum procalcitonin concentrations in patients with sepsis and infection Lancet 1993 341 515 518 10.1016/0140-6736(93)90277-N 8094770 PMC7141580

[B9] Sarı O Aydoğan U Alanbay İ Atacan İ Ercan CM Mesten Z Sağlam K Gebelerde Asemptomatik Bakteriüri Sıklığı Konuralp Tıp Dergisi 2011 3 9 1321646326

[B10] Nawas B Krammer I Shah PM Procalcitonin in diagnosis of severe infections Eur J Med Res 1996 1 331 333 9364034

[B11] Paccolat C Harbarth S Courvoisier D Irion O De Tejada BM Procalcitonin levels during pregnancy, delivery and postpartum J Perinat Med 2011 39 679 683 21834611 10.1515/jpm.2011.082

[B12] Assumma M Signore F Pacifico L Rossi N Osborn JF Chiesa C Serum procalcitonin concentrations in term delivering mothers and their healthy offspring: a longitudinal study Clin Chem 2000 46 1583 1587 11017935

[B13] Montagnana M Lippi G Albiero A Scevarolli S Salvagno GL Franchi M Guidi GC Procalcitonin values in preeclamptic women are related to severity of disease Clin Chem Lab Med 2008 46 1050 1051 18624624 10.1515/CCLM.2008.199

[B14] Gulec UK Ozgunen FT Guzel AB Buyukkurt S Seydaoglu G Urunsak IF Evruke IC An analysis of C-reactive protein, procalcitonin, and D-dimer in pre-eclamptic patients Am J Reprod Immunol 2012 68 331 337 10.1111/j.1600-0897.2012.01171.x 22783989

[B15] Leroy S Romanello C Galetto-Lacour A Bouissou F Fernandez-Lopez A Smolkin V Gurgoz MK Bressan S Karavanaki K Tuerlinckx D Leblond P Pecile P Coulais Y Cubells C Halevy R Aygun AD Da Dalt L Stefanidis CJ Borght TV Bigot S Dubos F Gervaix A Chalumeau M Procalcitonin is a predictor for high-grade vesicoureteral reflux in children: meta-analysis of ındividual patient data J Pediatr 2011 159 644 651 10.1016/j.jpeds.2011.03.008 21511275

[B16] Pecile P Miorin E Romanello C Falleti E Valent F Giacomuzzi F Tenore A Procalcitonin: a marker of severity of acute pyelonephritis among children Pediatrics 2004 114 249 254 10.1542/peds.114.2.e249 15286264

[B17] Küçük M Yavaşoğlu İ Kadıköylü G Bolaman Z Gebelik Ve Hematoloji Nobel Med 2011 7 10 17

[B18] Bedell SE Bush BT Erythrocyte sedimentation rate, from folklore to facts Am J Med 1985 78 1001 1009 10.1016/0002-9343(85)90224-4 4014259

[B19] Sacks GP Seyani L Lavery S Trew G Maternal C-reactive protein levels are raised at 4 weeks gestation Hum Reprod 2004 19 1025 1030 10.1093/humrep/deh179 14990546

